# On the estimation of heat-intensity and heat-duration effects in time series models of temperature-related mortality in Stockholm, Sweden

**DOI:** 10.1186/1476-069X-11-23

**Published:** 2012-04-10

**Authors:** Joacim Rocklov, Adrian G Barnett, Alistair Woodward

**Affiliations:** 1 Department of Public Health and Clinical Medicine, Epidemiology and Global Health, Umeå University, Umeå, Sweden; 2 Institute of Health and Biomedical Innovation & School of Public Health, Queensland University of Technology, Brisbane, Australia; 3 School of Population Health, University of Auckland, Auckland, New Zealand

## Abstract

**Background:**

We examine the effect of heat waves on mortality, over and above what would be predicted on the basis of temperature alone.

**Methods:**

Present modeling approaches may not fully capture extra effects relating to heat wave duration, possibly because the mechanisms of action and the population at risk are different under more extreme conditions. Modeling such extra effects can be achieved using the commonly left-out effect-modification between the lags of temperature in distributed lag models.

**Results:**

Using data from Stockholm, Sweden, and a variety of modeling approaches, we found that heat wave effects amount to a stable and statistically significant 8.1-11.6% increase in excess deaths per heat wave day. The effects explicitly relating to heat wave duration (2.0–3.9% excess deaths per day) were more sensitive to the degrees of freedom allowed for in the overall temperature-mortality relationship. However, allowing for a very large number of degrees of freedom indicated over-fitting the overall temperature-mortality relationship.

**Conclusions:**

Modeling additional heat wave effects, e.g. between lag effect-modification, can give a better description of the effects from extreme temperatures, particularly in the non-elderly population. We speculate that it is biologically plausible to differentiate effects from heat and heat wave duration.

## Background

Heat stress can lead to fatal consequences due to: dehydration; increased cardiovascular stress; kidney dysfunction; and electrolyte disorders [1,2]. At a population level, many studies show mortality tends to rise with higher temperatures [3]. Two approaches are generally used to quantify excess mortality: studies that focus exclusively on heat waves (so called episode studies); and studies that use time series to estimate the effects of temperature on mortality by averaging over hot days and heat waves. Heat waves are commonly referred to as a period of extreme heat stress relative to the normal climate, although the exact definition varies according to the number of consecutive days of heat, temperature variable(s) and heat threshold. Many time series studies, assuming the association between temperature and mortality is non-linear, report associations between heat and mortality that are immediate or delayed by up to a week [4,5]. However, the validity of this approach is challenged by research that reports an additional effect for heat waves [6]. Other studies have reported that heat-related mortality is sensitive to the duration of heat waves regardless of the intensity of the ambient heat (e.g. in France during the 2003 heat wave [7]). A number of studies have since explored additional heat waves effects with respect to their timing, intensity, duration and location [6,8-13]. All studies found statistically significant additional risks that may relate to the duration of heat waves and the cumulative extreme heat exposure. The main differences between the studies were the models used to estimate the heat-mortality relationship and the location.

The reason why the overall temperature-mortality relationship may not fully explain effects during heat waves is because the physiological effects of high temperatures and heat waves are different. For example, cumulative heat stress during a prolonged heat wave is more likely to cause dehydration. Cumulative heat stress is also more strongly related to cardiovascular deaths [8,13]. Differences in age stratified relative risks to heat and to heat waves have shown that the population at risk may differ, with the middle aged population potentially at the highest risk during heat waves [8,13].

We explain why additional heat wave effects are not perfectly captured in models of temperature-related mortality, and illustrate the estimation of additional heat wave effects using empirical data. We also explore how the additional heat wave effect is dependent on the complexity of temperature-mortality model. Using prior studies and our own results, we argue that future studies should evaluate potential additional effects from heat waves by decomposing heat exposure into a temperature term and an added heat wave term. Increasing model complexity will not suffice if there is no differentiation between heat wave days and non-heat wave days.

### Modelling weather-related mortality

Time series methods based on daily data have been developed and applied in studies of the short-term health effects of environmental factors like air pollution and weather [14,15]. Models often include lagged effects of exposure and adjustment for potential mortality displacement [14,16,17]. Studies have also explored: the use of non-linear functions to adjust for confounding (e.g., season), allowing non-linear exposure-response relationships, and the use of model fitting criteria [17,18].

### Why additional heat wave effects?

**Figure 1:**
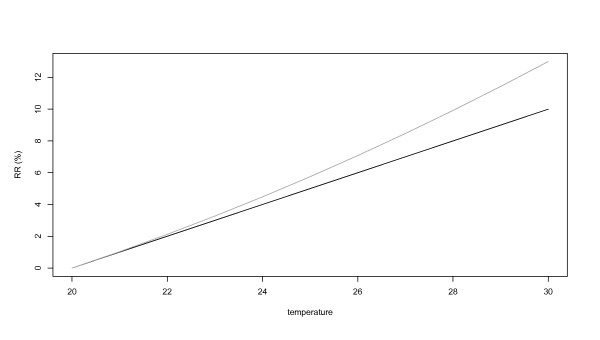
The fit of a linear (black) and a non-linear (grey) curve to data.

Additional heat wave effects may appear as an artefact if models employ an overly simple exposure-response relationship that does not adequately capture the non-linear effect with higher temperatures. In this case the additional effect is related directly to the potential misspecification of the effect. This is illustrated in Figure 1 where a linear relationship and a non-linear relationship are fitted to explain the relative risk (RR). Assuming that the factual relationship is wrongly assumed linear, an additional heat wave effects would try to compensate the difference between these two curves. This type of misspecifications can be addressed by allowing a more flexible non-linear exposure response relationship.

Additional heat wave effects can also arise due to cumulative heat stress. It is tempting to believe that cumulative stress can be estimated just like other delayed effects of heat exposure effects, e.g. by distributed lag models. This is not the case. Distributed lag models allow temperature a few days or weeks prior to day *t* to affect mortality on day *t*, and so are perfectly able to capture delayed effects of temperature. However, distributed lag models assume the delayed effects are related to the temperature on day *t* only. This means that delayed lag effects are independent of other lag days, for example, days *t-1* and *t-2*. They cannot model health effects caused by the temperature being above a heat threshold for a number of consecutive days. Thus, the distributed lag effect at a certain day of the heat wave does not estimate the effects relating to persistent heat stress. In order to estimate the effects relating to several days consecutive heat exposure above a certain threshold one would need to include non-linear interactions (effect-modifications) between the temperature lag effects. Here the non-linearity relates to the fact that the additional heat wave effects are thought to appear above some extreme temperature threshold. Thus, short-term cumulative stress of extreme heat can be described by lag effect-modification.

For illustration we consider the simple case where the effects of temperature are assumed to be dichotomous according to a threshold of the 98^th^ percentile of the temperature distribution.

(1) $$T_{t}=\{{}_{0\;else}^{1\;if\;temperature\;at\;time\;t>98th\;percentile}$$

The effect of extreme temperature (T) on mortality up to lag 3 is then in a non-constrained distributed lag model be estimated in a regression model:

(2) $$mortality_{t}\;\sim \;Poisson\left(mean_{t}\right)$$

(3) $$log\left(mean_{t}\right)=intercept+T_{t}+T_{t-1}+T_{t-2}+confounders$$

This model assumes that the effect of extreme temperature on day *t* is independent of whether the temperatures on day *t*–1 and *t*–2 are also extreme. This would not be the case if there are additional effects from persistent periods of extreme heat. Then, mortality on day *t* is also conditional on the temperatures on day *t–1* and *t–2*. To capture this in a regression model we can add two- and three-way interactions of the lag terms. In the model below we assume there are additional conditional relationships only between lags that are consecutive in time (e.g. an extended period without relief from the extreme temperatures),

(4) $$log\left(mean_{t}\right)=intercept+T_{t}+T_{t-1}+T_{t-2}+T_{t}\times T_{t-1}+T_{t}\times T_{t-1}\times T_{t-2}+confounders$$

When the number of lags studied is large (or if heat waves are long) this type of model will require a large number of interaction terms to be estimated, resulting in collinearity (similarly to the unconstrained distributed lag model). To avoid this we can add all the interaction terms together to create one variable denoting extended heat wave periods. This binary indicator variable is set to 1 if at least two or more proceeding days exceed the extreme temperature threshold, and 0 otherwise. We refer to this variable as a heat wave indicator variable (HWI). Note that our HWI is equal to *T*_*t*_ *× T*_*t-1*_, but it cannot be separated from days where $T_{t}\times T_{t-1}\times T_{t-2}=1$. Thus, the HWI variable describes the two-way interaction that was previously described in model (b). A new model including this variable can be expressed as,

(5) $$log\left(mean_{t}\right)=intercept+T_{t}+T_{t-1}+T_{t-2}+HWI_{t}+confounders_{t}$$

The model (c) with the HWI will not differentiate between two and three way interactions (e.g. two and three days of heat wave), as it assumes the additional effect is the same independent of the length of the heat wave. We might, however, have reason to suspect that longer heat waves are associated with larger additional effects. In this case we can construct a new variable with distinct values for shorter and longer heat waves. We refer to this variable as a heat wave duration variable (HWD). In the case where we use only 3 lag days we define HWD as to equal $T_{t}\times T_{t-1}+T_{t}\times T_{t-1}\times T_{t-2}$. In this example the HWD variable takes the values 0, 1, and 2 depending on how many consecutive days of extreme temperatures there are in before day *t*. The effect of heat wave duration can be estimated as a linear or non-linear function, or as a factor variable directly yielding the interactions. A non-linear function would be sensible if, for example, the effect of duration increased quickly and then remained high. Assuming linearity we get the regression model:

(6) $$log\left(mean_{t}\right)=intercept+T_{t}+T_{t-1}+T_{t-2}+HWD_{t}+confounders_{t}$$

The model (d) estimates the effects of the duration of heat waves as a linear function avoiding potential collinearity induced by explicitly including many lag interaction variables. The models above assume a dichotomous exposure-response relationship for temperature, and that effects of temperature and heat waves are not extended over more than 3 lag days. However, the same principle can be used when fitting a distributed lag model and a larger number of lag days.

### Model choice and additional heat wave effects

While recent studies found significant additional effects from heat waves, particularly in cold climates (such as the northern parts of the US and Sweden [9,19]), the size of this effect has been estimated using different approaches [9,10,12,13]. The main differences were: i) the complexity of the model used for the exposure-response relationship between temperature and mortality; ii) allowing for spatial heterogeneity in the additional heat wave effect; iii) allowing for heterogeneity in the additional heat wave effects between population sub-groups.

The study presenting the smallest additional effect from heat waves found the size of the effects to be almost negligible when the exposure-response relationship was allowed a very flexible parameterization using two dimensional cubic spline functions for temperature and lag day and modeling the main effect in a first stage model and the effect-modification in a second stage [12]. However, the estimates were for all-cause mortality independent of geographical differences in U.S. cities, while there is evidence that heat wave effects may be very sensitive to age, cause of death and location [8,9,13,19]. In particular, additional heat wave effects were negligible in the southern US, and large in the north east of the US [1,9,19].

Two studies estimated the overall temperature-mortality relationship using non-linear distributed lags and two-dimensional spline functions, and then tested the sensitivity of this parameterization [10,20]. Other studies used less complex linear and/or non-linear exposure response relationships, with a small number of lag days of between 1 and 3 [6,8,9,13,21]. The lag days in these studies were chosen according to prior literature and model fit criteria.

Bobb et al. argued against fitting one model across a range of climates (using the same degrees of freedom, splines and temperature measures), as they found that in most cities there were two or more models with a similar fit to the data [19]. Interestingly, after averaging over many different models they found larger effects of temperatures on heat wave days compared to non-heat wave days [19].

### Estimation of additional heat wave effects in Stockholm, Sweden

## Methods

We applied a non-linear distributed lag model to the effects of temperature in Stockholm County, Sweden, on total mortality during the years 1990–2002. Table 1 describes the basic characteristics of the data. We used daily maximum temperature as the predictor of daily mortality, adjusting for long-term time trends, seasonality, days of the week and national holidays in a non-linear distributed lag framework. This is model (1).

**Table 1:** Descriptive statistics for daily deaths and environmental variables in Stockholm County, 1990–2002, per season

Daily deaths, all non-injury causes, all ages	40 ± 7.2
Ages 45–79	18 ± 4.6
Ages 80+	20 ± 5.3
Mean temperature (˚C)	7.5 ± 7.6 (7 %)
range	–15.7, 26.4
Maximum temperature (˚C)	10.5 ± 8.6 (7 %)
range	–13.4, 33.5
Maximum temperature 98^th^ percentile (˚C)	27.5 C

To estimate additional heat wave effects (e.g. lag effect-modifications) we incorporated the variables HWI and HWD as described in a previous section. The frequencies of the HWD and HWI variables are in Table 2. Heat waves were defined as at least two days with maximum daily temperature above the 98^th^ percentile. So, on the second day with a temperature over the threshold, the variable was 1, and on the 7th consecutive day the variable was 6. The first day of temperature above the threshold was set to 0 corresponding to no accumulated heat effects. We first fitted the effect of heat wave duration as a smooth function (penalized spline with 4 degrees of freedom), but as it showed an approximately linear association we fitted a linear term. The model with the parameter for duration of heat waves, HWD, is model (2). The duration term estimates the effect-modification of cumulative lag terms above the 98^th^ percentile indirectly, with the prior assumption that the length of the heat wave period can influence the mortality response.

**Table 2:** Values and frequencies taken by the heat wave duration and the heat wave indicator variable

**Variable**	**Heat wave duration (HWD)**								**Heat wave indicator (HWI)**	
Value	0	1	2	3	4	5	6	7	0	1
Frequency	4697	23	11	7	4	3	2	1	4697	51

In model (3) we estimated the additional effects from heat waves (maximum temperature above 98^th^ percentile for at least two days) using an indicator variable, HWI. This model does not estimate additional effects due to heat wave duration explicitly, but the average additional excess mortality during the heat wave periods. The heat wave indicator estimates the effect modification of lag terms above the 98^th^ percentile assuming all days above this threshold are equally contributing to the mortality response.

As equations the three models are:

(7) $$mortality_{t}\;\sim \;Poisson\left(mean_{t}\right)$$

(8) $$log\left(mean_{t}\right)=intercept+S\left(temperature_{t}\text{,}\;lag.df\text{,}\;var.df\right)+S\left(time_{t}\text{,}\;var.df=6\;per\;year\right)+DOW_{t}+HD_{t}$$

(9) $$log\left(mean_{t}\right)=intercept+S\left(temperature_{t}\text{,}\;lag.df\text{,}\;var.df\right)+S\left(time_{t}\text{,}\;var.df=6\;per\;year\right)+DOW_{t}+HD_{t}+HWD_{t}$$

(10) $$log\left(mean_{t}\right)=intercept+S\left(temperature_{t}\text{,}\;lag.df\text{,}\;var.df\right)+S\left(time_{t}\text{,}\;var.df=6\;per\;year\right)+DOW_{t}+HD_{t}+HWI_{t}$$

Here *t* is the time in days, *S* is a cubic spline function, the spline function of temperature is two dimensional with lag degree of freedom given by *lag.df* and variable degree of freedom given by *var.df*. HWD is a linear variable denoting the day of the heat waves, HWI is an indicator variable for heat waves. time_t_ estimates trends and seasonal changes using a spline with 6 degrees of freedom per year (78 degrees of freedom in total), *DOW* denotes the day of week, and *HD* denotes national holidays.

We used the “dnlm” package in R [22]. We tested degrees of freedom for temperature from 3 to 8. The lagged effects of temperature were examined over 20 days and allowed 2, 4 or 6 degrees of freedom. The Akaike Information Criterion (AIC) was used to judge the optimal degrees of freedom.

We tested for differences by age through studying the groups: all ages; ages 80 years; and ages between 45–79 years.

We calculated the variation inflation factor as VIF = 1/(1–R-squared) to assess if the variance estimation was inflated through multi-collinearity introduced by having both temperature and heat wave terms in the same model. The variation inflation factor (VIF) of the duration variable and temperature was estimated to 1.048, and for the heat wave indicator variable to 1.058. So collinearity was not a concern for model 2 or 3.

## Results

**Figure 2:**
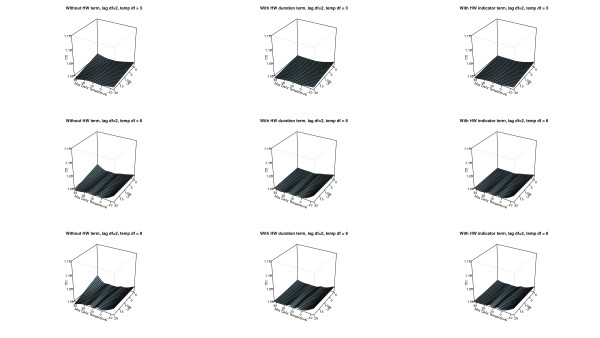
**The distributed non-linear lag (2*df)*surface relating to a daily max temperature of 12 C.** The models to the left have no heat wave variables (1), the models in the middle include a heat wave duration variable (2), and the models to the right include a heat wave indicator variable (3). The figure shows parameterizations using 2 *df* for the lags and 3, 6, and 8 *df* for the variable in the rows starting from the top respectively.

**Figure 3:**
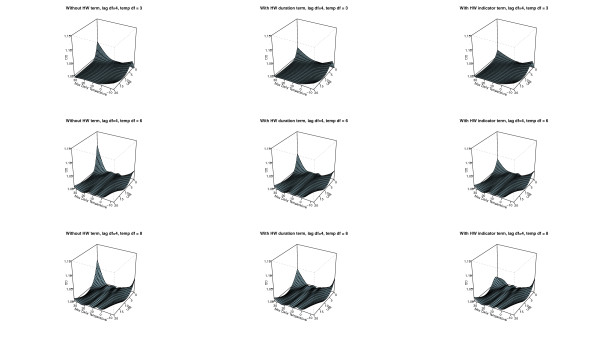
**The distributed non-linear lag (4*df)*surface relating to a daily max temperature of 12 C.** The models to the left are without additional heat wave variables (1), the models in the middle include a heat wave duration variable (2), and the models to the right include a heat wave indicator variable (3). The figure shows parameterizations using 4 *df* for the lags and 3, 6, and 8 *df* for the variable in the rows starting from the top respectively.

**Figure 4:**
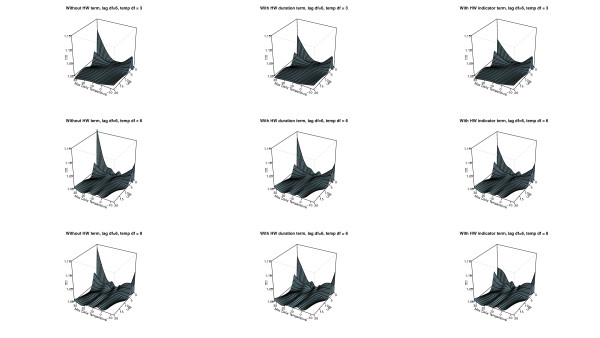
**The distributed non-linear lag (6*df)*surface relating to a daily max temperature of 12 C.** The models to the left are without additional heat wave variables (1), the models in the middle include a heat wave duration variable (2), and the models to the right include a heat wave indicator variable (3). The figure shows parameterizations using 6 *df* for the lags and 3, 6, and 8 *df* for the variable in the rows starting from the top respectively.

The AIC values associated with each model for mortality in all ages are in Table 3. Overall the models including additional heat wave variables (model 2 and 3) gave a better fit to the data compared with model 1 for the same degrees of freedom. The two best fits are a simple parameterization using an indicator variable or a heat wave duration variable with *3 df* for temperature and *2 df* for the lagged effects as main effect. These models resulted in a small marginal heat effect on non-heat wave days (Figure 2), while more flexible parameterizations for the main temperature effect estimated a larger effect (Figures 2 and 3). However, with increasing complexity of the main temperature-mortality relationship the model fit decreased and the bendiness of the estimated two dimensional spline functions increased considerably as can be seen in Table 3 and Figures 2–4. Figures 2–4 also show that models including additional heat wave effects predict a lower mortality increase for high temperatures at short lags compared with the model with no heat wave variable. The effect of heat on lag 0 is then higher in the model without an additional heat wave effect. This shows that the model without an additional heat wave effect would estimate higher marginal excess mortality on days with high temperatures that were not part of the heat wave compared to the model with the additional heat wave effect. It appears that models with an explicit allowance for additional heat wave effects do a better job of describing the exact distribution of heat-related deaths.

**Table 3:** AIC values for the three models using 3 to 8 degrees of freedom for temperature and 2 to 6 degrees of freedom for lag, Stockholm, 1990–2002 in all ages

***df* Temperature Spline**	**No heat wave variable (model 1)**	**With heat wave duration variable (model 2)**	**With heat wave indicator variable (model 3)**
	*df* Lag Spline = 2		
3	20052	20044	20040
4	20056	20047	20044
5	20059	20051	20048
6	20058	20051	20047
7	20062	20055	20052
8	20063	20057	20054
	*df* Lag Spline = 4		
3	20054	20049	20047
4	20055	20052	20051
5	20060	20059	20057
6	20061	20061	20059
7	20061	20061	20059
8	20067	20068	20065
	*df* Lag Spline = 6		
3	20061	20056	20053
4	20065	20063	20061
5	20075	20073	20071
6	20077	20077	20075
7	20082	20081	20079
8	20089	20089	20086

In the age group 80 years and above the inclusion of additional heat wave variables did not improve the model fit (Table 4). In the age group 45–79 years, however, the inclusion of an additional heat wave variable improved the model fit according to the AIC (Table 5), while it is not possible to distinguish between the model including the HWD (model 2) and HWI (model 3) in terms of model fit.

**Table 4:** AIC values for the three models using 3 to 8 degrees of freedom for temperature and 2 to 6 degrees of freedom for lag, Stockholm, 1990–2002 in ages 80 years of age and above

***df* Temperature Spline**	**No heat wave variable (model 1)**	**With heat wave duration variable (model 2)**	**With heat wave indicator variable (model 3)**
	*df* Lag Spline = 2		
3	17962	17961	17957
4	17966	17965	17961
5	17970	17969	17965
6	17971	17970	17966
7	17974	17972	17968
8	17977	17975	17971
	*df* Lag Spline = 4		
3	17958	17960	17958
4	17959	17961	17961
5	17967	17969	17968
6	17971	17973	17972
7	17977	17979	17977
8	17982	17984	17983
	*df* Lag Spline = 6		
3	17958	17959	17957
4	17961	17963	17963
5	17972	17974	17974
6	17980	17981	17981
7	17989	17991	17990
8	17997	17999	17998

**Table 5:** AIC values for the three models using 3 to 8 degrees of freedom for temperature and 2 to 6 degrees of freedom for lag, Stockholm, 1990–2002 in ages 45 to 79 years of age

***df* Temperature Spline**	**No heat wave variable (model 1)**	**With heat wave duration variable (model 2)**	**With heat wave indicator variable (model 3)**
	*df* Lag Spline = 2		
3	17410	17405	17406
4	17415	17409	17410
5	17416	17412	17412
6	17416	17412	17413
7	17417	17415	17415
8	17419	17417	17417
	*df* Lag Spline = 4		
3	17418	17413	17413
4	17423	17418	17419
5	17428	17424	17425
6	17430	17428	17428
7	17426	17426	17426
8	17434	17434	17434
	*df* Lag Spline = 6		
3	17426	17421	17421
4	17435	17430	17430
5	17443	17440	17440
6	17448	17447	17446
7	17448	17448	17447
8	17456	17457	17456

**Figure 5:**
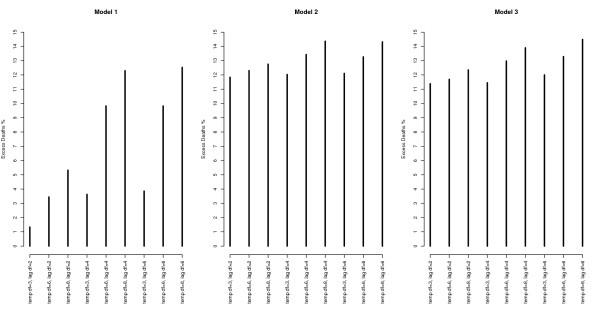
**The excess mortality predictions for day 4 of a heat wave with maximum daily temperatures at 31 C as compared to 2 C.** The predictions include the 0–3 lagged effects over a range of different degrees of freedom of the main temperature-mortality relationship and: i) from a model with no added heat wave effect (model 1); ii) from a model with a heat wave duration parameter (model 2); iii) and from a model with the added heat wave effect as a dummy variable (model 3).

**Figure 6:**
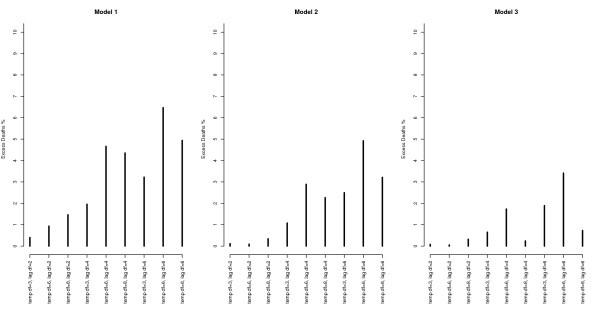
**The excess mortality predictions for a single hot day (non-heat wave day) for maximum daily temperatures at 31 C as compared to 26 C.** The predictions include only the lag 0 effect over a range of different degrees of freedom of the main temperature-mortality relationship and: i) from a model with no added heat wave effect (model 1); ii) from a model with a heat wave duration parameter (model 2); iii) and from a model with the added heat wave effect as a dummy variable (model 3).

Figures 5 and 6 show the effects of a 5 unit increase in maximum daily temperature from 26 C to 31 C (the heat wave threshold is 27.5 C). Figure 5 shows the excess mortality predictions for day 4 of a heat wave (including the 0–3 day lagged effects) while varying the degrees of freedom of the main temperature-mortality relationship, and showing the results for three different models. In broad terms, the heat wave predictions are similar with more complex model fits, but models 2 and 3 (which include terms for heat wave effects) predict higher excess mortality for the less complex temperature-mortality parameterizations. However, even for complex parameterizations, the model without an additional heat wave effect predicts a few percent lower excess mortality compared to the two heat wave models.

Figure 6 shows the corresponding marginal excess mortality predictions for a single hot day (non-heat wave day; lag 0 only). The model without the added heat wave effect (model 1) predicts higher mortality on non-heat wave days compared to the models that differentiate between heat wave and non-heat wave days (model 2 and 3). Thus, model 1 (without the additional heat wave effect) may over-estimate the effect of heat on non-heat wave days, while it appears to under-estimate the effect from heat on heat wave days. Models 2 or 3 do better in this sense, differentiating the effects between heat wave days and non-heat wave days through the variables capturing the effect modification between the lags of temperature. The more complex parameterizations of model 2 and 3 strongly indicate over-fitting in graphical examinations, whilst having better AIC values.

The optimal fit of model 3 estimated a relative risk of 1.112 (95% confidence interval of 1.050, 1.176) associated with heat wave days (95% confidence interval of 1.050, 1.176). The effects ranged between 1.116 and 1.083, and all estimates were statistically significant at the 5% level as is shown Table 6.

**Table 6:** Relative risks (RRs) and confidence intervals (CI; 95%) associated with heat waves

	***Heat wave duration variable (model 2; unit: days of duration)***		***Heat wave indicator variable (model 3; unit: heat wave = {yes, no})***	
*df* Temperature Spline	*df* Lag Spline = 2			
	RR	CI	RR	CI
3	1.037	1.014, 1.060	1.112	1.050, 1.176
4	1.039	1.015, 1.063	1.116	1.053, 1.183
5	1.038	1.013, 1.062	1.114	1.050, 1.182
6	1.038	1.013, 1.063	1.114	1.049, 1.182
7	1.038	1.013, 1.063	1.114	1.048, 1.183
8	1.037	1.011, 1.062	1.111	1.045, 1.180
	*df* lag spline = 4			
3	1.032	1.008, 1.056	1.100	1.034, 1.169
4	1.028	1.002, 1.054	1.088	1.018, 1.162
5	1.026	0.998, 1.053	1.086	1.011, 1.165
6	1.023	0.994, 1.051	1.081	1.003, 1.165
7	1.022	0.992, 1.051	1.087	1.005, 1.174
8	1.020	0.990, 1.051	1.088	1.004, 1.179
	*df* lag spline = 6			
3	1.032	1.008, 1.056	1.102	1.037, 1.172
4	1.028	1.002, 1.054	1.090	1.020, 1.165
5	1.025	0.998, 1.053	1.088	1.013, 1.168
6	1.023	0.994, 1.052	1.083	1.005, 1.167
7	1.022	0.993, 1.052	1.088	1.006, 1.176
8	1.019	0.989, 1.050	1.088	1.003, 1.179

We did not assess the estimates’ sensitivity to the parameterization (*df*) of long-term time trends, as this appears to have less influence on the heat wave effect [12,19].

## Discussion

Estimates of additional heat wave effects in models of temperature-related mortality can be interpreted as a constrained form of non-linear effect-modifications between lags of high temperature (or, similarly lag interactions). This can explain why such effects have been found to significantly contribute to additional deaths during heat waves in previous studies of temperature related mortality, over and above the effects of temperature overall. This dispels the widespread belief that such effects are incorporated through distributed lag models. From a mechanistic perspective including additional effects from heat waves are supported through the physiological stress incurred by cumulative exposure being potentially different from the stress from shorter periods of extreme heat, and can also result in differences in the population at risk such as contrasting susceptibility with age [8-10,13,19].

We found the additional heat wave effects were more important in middle age populations and the elderly compared to the very elderly. We found the size of the heat wave effect depended on the complexity of the main temperature-mortality parameterization, more specifically on the *df* used for modeling non-linearity of temperature and lagged effects. This indicates that there is, not surprisingly, some overlap between the effects of hot days and the effects of heat waves. Our results show, however, that there is also likely to be an independent extra effect of heat waves that is not captured by hot days, e.g. an effect modification. Differences in the temperature-mortality parameterization probably explain many of the differences between the conclusions of recent studies on the effects of heat waves [6,8,9,11-13,19,21] In our example we found that a simple model for the temperature-mortality association was better than a complex model. The additional heat wave effects are substantial and important to account for in order not to underestimate mortality risks during heat wave days. We conclude that it is important not to over fit the data by using too complex non-linear lag parameterizations, and that simple parameters for the additional effects of heat waves are useful. However, the more complex parameterizations appear to better capture effects and the high end. It appear it may not be reasonable to distribute the degrees of freedom uniformly over the temperature and lag scales as such assumption gave rise to over-fitting in regions of the temperature and lag scale where the relationship is not very complex. Overall, model fit improved as the complexity of the model and flexibility of the splines were reduced. These simpler models also had larger additional heat wave effects, as reported elsewhere [6,8,9,11-13,19,21].

Some studies have used two-stage models to describe the effects from temperature using distributed lag non-linear models in the first stage and the additional effect associated with heat waves in a second step [10,12]. We note, however, that this deviates from the conventional framework for modeling of effect-modifications, and that it can potentially affect the estimates of additional heat wave effects downward.

We achieved a better model fit using an indicator variable for heat waves rather than the duration variable when studying all ages; nevertheless, models with the duration variable performed better than the models without additional heat wave components, and similarly well in the age group 45–79 years of age.

## Conclusions

We conclude that it is important to continue to explore the magnitude of additional heat wave effects in future studies of temperature-related mortality, e.g. temperature lag effect-modifications. It appear also important to fit models that are location sensitive in the parameterization (choice of *df*), as well as in the evaluation of potential additional heat wave effects. Fitting a complex distributed lag non-linear model may reduce the heat wave signal and over-estimate mortality on non-heat wave days, compared to a model including a heat wave term. It is important to be able to differentiate between extended periods of heat and single days of extremely high temperatures, since several recent studies have shown that duration of heat exposure is related to mortality risk. Increasing the accuracy of heat-wave mortality models will assist public health authorities to direct preventive actions when they are most needed. Future studies should continue to study and identify potential differences in the population at risk to heat and heat waves, as well as describe the mechanistic differences.

## Competing interests

The authors declare that they have no competing interests.

## Authors’ contributions

JR designed the study, analysed the data, interpreted the results and drafted the manuscript. AB helped design the study, advised in the analysis of data, interpreted the results and helped draft the paper. AW interpreted the results, advised in the analysis and helped draft the paper.

## References

[B1] Kilbourne EM The spectrum of illness during heat waves Am J Prev Med 1999 16 4 359 360 10493296 10.1016/s0749-3797(99)00016-1

[B2] Parsons K Human Thermal Environments. The effects of hot, moderate and cold temperatures on human health, comfort and performance 2003 2 CRC Press, New York 2003

[B3] Kovats RS Hajat S Heat stress and public health: a critical review Annu Rev Public Health 2008 29 41 55 10.1146/annurev.publhealth.29.020907.090843 18031221

[B4] Baccini M Biggeri A Accetta G Kosatsky T Katsouyanni K Analitis A Anderson HR Bisanti L D’Ippoliti D Danova J Heat effects on mortality in 15 European cities Epidemiol 2008 19 5 711 719 10.1097/EDE.0b013e318176bfcd 18520615

[B5] Braga AL Zanobetti A Schwartz J The time course of weather-related deaths Epidemiol 2001 12 6 662 667 10.1097/00001648-200111000-00014 11679794

[B6] Hajat S Armstrong B Baccini M Biggeri A Bisanti L Russo A Paldy A Menne B Kosatsky T Impact of high temperatures on mortality: is there an added heat wave effect? Epidemiol 2006 17 6 632 638 10.1097/01.ede.0000239688.70829.63 17003686

[B7] Fouillet A Rey G Laurent F Pavillon G Bellec S Guihenneuc-Jouyaux C Clavel J Jougla E Hemon D Excess mortality related to the August 2003 heat wave in France Int Arch Occup Environ Health 2006 80 1 16 24 10.1007/s00420-006-0089-4 16523319 PMC1950160

[B8] Anderson BG Bell ML Weather-related mortality: how heat, cold, and heat waves affect mortality in the United States Epidemiol 2009 20 2 205 213 10.1097/EDE.0b013e318190ee08 PMC3366558 19194300

[B9] Anderson GB Bell ML Heat waves in the United States: mortality risk during heat waves and effect modification by heat wave characteristics in 43 U.S. communities Environ Health Perspect 2011 119 2 210 218 21084239 10.1289/ehp.1002313 PMC3040608

[B10] Barnett AG Hajat S Gasparrini A Rocklov J Cold and heat waves in the United States Environ Res 2012 112 218 224 22226140 10.1016/j.envres.2011.12.010

[B11] D'Ippoliti D Michelozzi P Marino C de’Donato F Menne B Katsouyanni K Kirchmayer U Analitis A Medina-Ramon M Paldy A The impact of heat waves on mortality in 9 European cities: results from the EuroHEAT project Environ Health 2010 9 37 10.1186/1476-069X-9-37 20637065 PMC2914717

[B12] Gasparrini A Armstrong B The impact of heat waves on mortality Epidemiol 2011 22 1 68 73 10.1097/EDE.0b013e3181fdcd99 PMC3324776 21150355

[B13] Rocklov J Ebi K Forsberg B Mortality related to temperature and persistent extreme temperatures: a study of cause-specific and age-stratified mortality Occup Environ Med 2011 68 7 531 536 10.1136/oem.2010.058818 20962034

[B14] Dominici F Time-series analysis of air pollution and mortality: a statistical review Res Rep Health Eff Inst 2004 123 3 27 discussion 29–33 15757000

[B15] Zeger SL A Regression-Model for Time-Series of Counts Biometrika 1988 75 4 621 629 10.1093/biomet/75.4.621

[B16] Schwartz J The distributed lag between air pollution and daily deaths Epidemiol 2000 11 3 320 326 10.1097/00001648-200005000-00016 10784251

[B17] Zanobetti A Wand MP Schwartz J Ryan LM Generalized additive distributed lag models: quantifying mortality displacement Biostatistics 2000 1 3 279 292 10.1093/biostatistics/1.3.279 12933509

[B18] Peng RD Dominici F Louis TA Model choice in time series studies of air pollution and mortality J R Statist Soc A 2006 169 25

[B19] Bobb JF Dominici F Peng RD A Bayesian model averaging approach for estimating the relative risk of mortality associated with heat waves in 105 U.S. cities Biometrics 2011 67 4 1605 1616 10.1111/j.1541-0420.2011.01583.x 21447046 PMC3128186

[B20] Sattar A Rubessa M Di Francesco S Longobardi V Di Palo R Zicarelli L Campanile G Gasparrini B The influence of gamete co-incubation length on the in vitro fertility and sex ratio of bovine bulls with different penetration speed Reprod Domest Anim 2011 46 6 1090 1097 10.1111/j.1439-0531.2011.01791.x 21535238

[B21] Rocklov J Forsberg B The effect of temperature on mortality in Stockholm 1998–2003: a study of lag structures and heatwave effects Scand J Public Health 2008 36 5 516 523 10.1177/1403494807088458 18567653

[B22] Gasparrini A Distributed Lag Linear and Non-Linear Models in R: The Package dlnm J Stat Softw 2011 43 8 1 20 22003319 PMC3191524

